# Buffalo Medical Journal

**Published:** 1853-06

**Authors:** 


### BUFFALO MEDICAL JOURNAL.

This excellent journal, for the monthly appearance of which we always look with interest, has just entered upon its ninth year. Dr. Austin Flint, who has conducted it with so much ability hitherto, is to be assisted in its editorial management hereafter by Dr. S. B. Hunt. We are glad to learn that "the present condition of the Journal is sufficiently prosperous to meet the views of its proprietors, and to secure its continuance."

